# Psychological experiences and consequences of classification and reclassification in competitive Para sport: a scoping review

**DOI:** 10.1186/s13102-026-02096-8

**Published:** 2026-09-22

**Authors:** Mehmet Aydoğan, Aygül Çağlayan Tunç

**Affiliations:** 1 Department of Exercise and Sport Sciences for Persons with Disabilities, Faculty of Sports Sciences, Istanbul Gelisim University, Istanbul, Türkiye; 2 Department of Physical Education and Sports Teaching, Faculty of Sports Sciences, Istanbul Aydin University, Istanbul, Türkiye

**Keywords:** Procedural justice, Psychological safety, Athlete well-being, Uncertainty, Identity, Trust, Career transition, Accessibility

## Abstract

**Background:**

Classification determines eligibility and sport class in competitive Para sport, yet its psychological consequences have not been synthesised separately from the technical classification literature. This review mapped athlete experiences and psychological consequences associated with classification, reclassification, declassification, and related classification decisions.

**Methods:**

A scoping review was conducted in accordance with JBI guidance and reported using PRISMA-ScR. Web of Science Core Collection and Scopus were searched from inception to 26 July 2026; PubMed/MEDLINE, Academic Search Ultimate, and OpenDissertations were searched from inception to 27 July 2026. Two reviewers independently completed title-and-abstract and full-text screening. Data were charted descriptively and synthesised using inductive thematic synthesis. Formal critical appraisal was not undertaken because the review was designed to map a heterogeneous evidence base rather than estimate effects or grade certainty.

**Results:**

The searches identified 2,658 records; 1,289 remained after duplicate removal. Twenty-four reports were sought for full-text retrieval, 22 were assessed, and 17 studies were included. The evidence comprised 10 primarily qualitative studies, four quantitative survey or cross-sectional studies, one mixed-methods study, and two participatory or multi-method evaluations. Seven themes were identified: process knowledge and communication; fairness, trust, and system integrity; uncertainty, control, and emotional consequences; identity, belonging, dignity, and social relations; satisfaction, acceptance, and perceived utility; motivation, self-efficacy, behavioural responses, and performance; and access, burden, and participation. The most disruptive accounts were reported when decisions were poorly explained, perceived as inconsistent, or associated with unexpected reclassification or loss of eligibility. Prospective and longitudinal evidence was scarce, and no included study prospectively evaluated a classification-specific support strategy.

**Conclusions:**

Classification was reported as both a technical assessment and an organisational and interpersonal process. Across the mapped evidence, transparent information, respectful communication, sport-specific procedures, credible review routes, and attention to access and transition were associated with trust, acceptance, and well-being. Classification-specific education, support, and referral approaches should be regarded as evidence-informed priorities for prospective evaluation, particularly around reclassification, ‘Not Eligible’ decisions, protest, appeal, and career transition.

**Supplementary Information:**

The online version contains supplementary material available at https://doi.org/10.1186/s13102-026-02096-8.

## Background

Classification is a central regulatory component of competitive Para sport. It determines whether an athlete has an eligible impairment, whether the impairment meets the minimum criteria for a particular sport, and the sport class in which the athlete may compete. The International Paralympic Committee (IPC) defines its purpose as enabling fair and meaningful competition by reducing the extent to which impairment determines sporting outcomes [1]. Classification therefore differs from selection or performance ranking: athletes are grouped according to the impact of impairment on the activities fundamental to their sport rather than their technical ability, training status, or competitive record [2, 3]. Classification is consequently an important component of Para sport, and its decisions may have substantial implications for eligibility, competitive opportunity, team composition, funding, and progression through an athlete pathway.

The scientific development of classification has concentrated largely on the validity, reliability, and sport specificity of impairment assessment. This work has strengthened evidence-based classification and reduced reliance on diagnosis alone [2, 3]. The 2025 IPC Classification Code continues that direction by seeking greater harmonisation across sports and by emphasising stakeholder confidence in classification procedures and outcomes [1]. Technical accuracy, however, does not fully account for how classification is encountered by the athlete. A process may satisfy formal rules while still being experienced as uncertain, opaque, intrusive, or difficult to contest. The psychological meaning of classification is especially important because the decision is not peripheral to participation; it can determine whether and how an athlete is permitted to pursue competitive sport.

Evidence from Para sport has identified classification as a distinctive organisational demand. Athletes have described late classification, unexpected class changes, limited communication, and perceived inaccuracies as sources of stress [4]. Studies of paraswimmers, wheelchair basketball players, and athletes with visual impairment have also documented concerns about subjectivity, fairness, restricted athlete voice, and trust in classifiers or competitors [5–7]. Interviews with athletes and classifiers showed that the quality of communication and the structure of the system influenced how classification was understood and experienced [8]. More recently, Para alpine skiers with visual impairment questioned the suitability of assessment procedures and reported experiences of exclusivity and distrust [9]. Psychological responses may therefore develop not only from class allocation but also from information deficits, interpersonal treatment, perceived procedural injustice, uncertainty about outcomes, and limited control over decisions with major sporting consequences.

The emotional and cognitive consequences reported in the literature include anxiety, frustration, confusion, dread, perceived loss of control, and distrust [6, 7, 10–13]. Bosma and Van Yperen examined functional classification as a potential competitive stressor in wheelchair basketball and found no direct association between functional class and competitive anxiety or self-efficacy [10]. This null association is informative because it suggests that the numerical class itself may not adequately explain athletes’ psychological responses; the broader literature instead points to how athletes appraise the process, including its predictability, communication, perceived fairness, and consequences. Autobiographical and qualitative accounts have described classification as an experience that can require athletes to repeatedly demonstrate or justify impairment, sometimes producing feelings of interrogation, objectification, or diminished dignity [7, 11]. The most detailed recent investigation of elite British athletes identified perceived systemic bias, psychological turmoil associated with uncertainty and lack of control, and a culture in which classification was difficult to discuss openly [12]. Clear communication and competent practice could, however, support more empowering experiences. A focused review must therefore include positive, negative, and ambivalent psychological responses rather than presuming that classification operates only as a stressor.

Reclassification and loss of eligibility may carry consequences that differ from an initial classification. Reclassification assigns an athlete to a sport class other than the one previously held, whereas declassification or a “Not Eligible” decision may remove access to a sport or level of competition. Such changes can alter competitive prospects, selection status, team roles, financial support, and perceptions of athletic competence. They may also disrupt the relationship between impairment, sporting identity, and belonging within the Para sport community. Peers described reclassification as making the losses associated with a degenerative condition newly visible [11], while research on Para-athlete retirement showed that externally imposed or poorly supported exits from sport can be particularly difficult to negotiate [13]. When a classification decision accelerates retirement or forces a change of sport, the psychological consequences may extend beyond immediate distress to identity disruption, reduced motivation, withdrawal, and uncertainty about future participation.

These concerns sit within a broader literature on mental health and well-being in elite Para sport. Para-athletes encounter many of the performance and organisational pressures experienced by other elite athletes, together with disability-specific demands related to accessibility, ableism, health management, equipment, and classification [14]. Socioecological research has shown that mental health outcomes are associated not only with individual characteristics but also with interpersonal and organisational factors, including psychological safety and the quality of the sport environment [15]. Qualitative work on elite Para-athlete well-being similarly highlights the influence of relationships, organisational practices, and the conditions under which athletes can exercise autonomy and seek support [16]. Classification should therefore be examined as a process embedded within the athlete’s social and organisational environment, rather than as a single technical event.

Lawson and Latimer-Cheung mapped the wider body of Para sport classification literature and systematically examined web-based resources [17]. Their scoping review established that the literature commonly addressed coach and classifier roles, athletes’ perspectives, classification instructions, and participation-related outcomes. Its scope was intentionally broad and did not organise the evidence around psychological mechanisms or distinguish the psychological implications of initial classification, reclassification, declassification, review status, protest, and appeal. Since that review, additional athlete-centred work has examined classification in visually impaired sport, Para alpine skiing, elite Para-athlete well-being, remote access to classification, and the experiences of elite British athletes [6, 9, 12, 16, 18]. The present review therefore advances the literature not simply by updating it, but by organising empirical evidence around psychological processes, classification stages, and reported consequences, while distinguishing observed experiences and associations from support strategies that remain to be prospectively evaluated.

A scoping review was appropriate because the available evidence spanned qualitative interviews, autobiographical accounts, observational research, quantitative studies, policy-oriented work, and studies in which classification formed one component of a broader investigation. Considerable variation was anticipated across sports, impairment groups, competition levels, classification systems, and national settings. Rather than estimating a single pooled effect, the review mapped the extent and characteristics of the evidence, clarified how psychological experiences and consequences had been conceptualised, and identified methodological and substantive gaps. The review followed the Joanna Briggs Institute methodological guidance for scoping reviews [19] and was reported in accordance with the PRISMA extension for scoping reviews [20].

The primary objective of this scoping review was to map and synthesise evidence concerning the psychological experiences and reported consequences of classification and reclassification among athletes participating in competitive Para sport. The review was guided by three questions: (1) what psychological experiences, processes, and reported outcomes have been examined across stages of the classification pathway; (2) how are these experiences represented across classification contexts, sports, impairment groups, competitive levels, and relevant interpersonal or organisational conditions; and (3) what methodological and substantive gaps remain, particularly for prospective research and athlete support? By separating psychological evidence from the broader technical literature, the review sought to provide a clearer basis for athlete-centred classification procedures and future research without treating untested support strategies as established effects.

## Methods

### Design and reporting framework

A scoping review design was selected because the available literature comprised heterogeneous qualitative, quantitative, mixed-methods, and multi-stakeholder evidence and because the purpose was to map how psychological experiences and consequences of Para sport classification had been conceptualised and studied rather than to estimate a pooled effect. The review was conducted in accordance with the JBI methodology for scoping reviews [21]. Reporting followed the Preferred Reporting Items for Systematic Reviews and Meta-Analyses extension for Scoping Reviews (PRISMA-ScR) [20]. Search methods were documented with reference to PRISMA-S [22], and the study-selection process was presented using the PRISMA 2020 flow diagram [23]. PERSiST guidance was also consulted to improve the clarity of reporting within a sport and exercise science context [24].

### Protocol and registration

The protocol was not prospectively registered in the Open Science Framework, PROSPERO, or another public registry, and no protocol article was published. Before formal screening began, the review team finalised an internal written review plan specifying the review questions, Population-Concept-Context framework, information sources, eligibility criteria, screening procedures, and core data-charting fields. The plan was not publicly time-stamped. During screening, minor operational clarifications and reasons for full-text exclusion were recorded in a review log; the core Population-Concept-Context framework, eligibility logic, screening procedure, and core data-charting domains remained unchanged. The database searches had been completed before formal screening, and the synthesis remained descriptive and inductive rather than effect-estimating. Because the internal plan was not prospectively registered or publicly time-stamped, it is not presented as a formally registered protocol.

### Eligibility criteria

Eligibility was defined using the JBI Population-Concept-Context framework. Sources were eligible when they contained original empirical evidence concerning competitive Para athletes’ experiences of, responses to, or consequences arising from a formal sport-classification process, or when they examined a psychological or behavioural construct in relation to an assigned sport class or another clearly defined classification exposure. No publication-date restriction was applied. No language filter was applied at the search stage. Reports in languages other than English were considered when an English-language title, abstract, or other source information provided sufficient detail for reliable screening and extraction. The review did not use a formal professional-translation or multilingual-reviewer procedure; the implications of this limitation are acknowledged below Table 1.

**Table 1:** Eligibility criteria structured according to the Population-Concept-Context framework

Domain	Inclusion criteria	Exclusion criteria
Population	Competitive Para athletes, including currently active athletes and former athletes whose sport participation or career transition was affected by a classification decision. Multi-stakeholder studies were eligible when athlete data were reported or could be extracted separately.	Non-athlete samples only, unless athlete findings were separately reported; participants involved solely in recreational disability sport or general physical activity without a competitive classification context.
Concept	Initial classification, reclassification, declassification, not-eligible decisions, review status, protest, appeal, intentional misrepresentation, or implementation/change of a classification system, together with psychological, emotional, cognitive, identity-related, motivational, behavioural, interpersonal, participation, or career consequences; or psychological and behavioural constructs examined in relation to assigned sport class or classification exposure.	Technical validity, reliability, biomechanics, physiology, impairment-performance modelling, test development, or minimum-impairment research without athlete-level psychological or experiential findings.
Context	Formal or structured classification within organised competitive Para sport, at any competitive level, in any country or sport.	Clinical disability classification, educational classification, legal disability determination, or general disability-sport participation unrelated to a formal sport-classification system.
Sources of evidence	Primary qualitative, quantitative, mixed-methods, survey, observational, or programme-evaluation studies; dissertations and theses containing original data.	Reviews, editorials, commentaries, conceptual papers, policy documents, expert consensus/Delphi studies without original athlete-experience data, and conference abstracts lacking sufficient extractable findings.

*PCC* Population-Concept-Context

### Information sources

The primary searches covered Web of Science Core Collection and Scopus as multidisciplinary citation databases, PubMed/MEDLINE for biomedical and rehabilitation literature, Academic Search Ultimate for multidisciplinary full-text coverage, and OpenDissertations for grey literature. Web of Science and Scopus were searched on 26 July 2026; PubMed/MEDLINE, Academic Search Ultimate, and OpenDissertations were searched on 27 July 2026. Database coverage extended from inception to the date of the search. SPORTDiscus, APA PsycINFO, and Embase were not searched because access was unavailable to the authors through institutional subscriptions or personal access routes available during the review period. Their omission may have resulted in database-related selection bias, particularly for sport-psychology and disability-focused literature, and the search should not be interpreted as exhaustive. To mitigate this limitation within the resources available, the review combined two multidisciplinary citation databases, a biomedical database, a multidisciplinary full-text database, and a dissertation database; used a deliberately broad classification search that did not require psychological terminology; conducted supplementary psychological sensitivity searches; and tested search sensitivity against six sentinel athlete-centred classification studies, all of which were retrieved by both Web of Science and Scopus.

### Search strategy

The search strategy was developed around two core concepts: (1) Para sport and Para athletes and (2) sport classification. Controlled vocabulary was used where available and was combined with free-text synonyms, spelling variants, truncation, and phrase searching. The primary strategy was intentionally broad and did not require a psychological term because pilot searches showed that several relevant athlete-experience studies did not identify psychological outcomes explicitly in their titles or abstracts. The syntax was translated for each database while preserving the same conceptual structure.

Supplementary psychological-focused searches were conducted as sensitivity checks in Web of Science (*n* = 346), Scopus (*n* = 372), and PubMed/MEDLINE (*n* = 187). Comparison with the primary-search library showed that these records were subsets of the corresponding broad searches and did not introduce additional unique eligible records. They were therefore not added to the PRISMA identification total as independent sources. Additional file 1 provides the database-specific search dates, platforms/interfaces, field restrictions, complete search strings, exact result counts, and the supplementary psychological sensitivity-search details.

### Study selection

All records were exported in RIS or NBIB format and combined in a master bibliographic library. Duplicate records were identified using a sequential rule-based procedure based on: (1) exact normalised DOI; (2) exact PMID; (3) exact normalised title and publication year; and (4) exact normalised long title when year metadata were missing or inconsistent. When duplicate records differed in completeness, the version containing the most complete DOI, abstract, journal, publication date, volume, issue, pagination, or article-number information was retained. Ambiguous duplicate groups were manually checked before screening.

Two reviewers (M.A. and A.Ç.T.) independently completed title-and-abstract screening against the eligibility criteria. Records were retained for full-text assessment whenever eligibility could not be determined confidently from the title and abstract. The same reviewers independently assessed the full texts. Disagreements at either stage were resolved through discussion, joint re-examination of the source, and application of the predefined operational criteria until consensus was reached. Reasons for exclusion were documented at the full-text stage.

The searches yielded 2,658 records. After removal of 1,369 duplicate records, 1,289 unique records underwent title-and-abstract screening. Twenty-four reports were sought for full-text retrieval. Full texts were sought through publisher websites, DOI resolution, PubMed Central, institutional repositories, and other legitimate repository records. Two reports could not be retrieved independently. Twenty-two full-text reports were assessed, five were excluded with documented reasons, and 17 studies were included in the final synthesis.

### Data charting

A structured data-charting form was developed in Microsoft Excel and tested on a subset of eligible studies before full charting. The form was refined iteratively when additional relevant fields emerged, while the core domains remained consistent with the review questions. M.A. completed the initial charting using the agreed form, and A.Ç.T. subsequently verified the completed charting against the source reports. Discrepancies or uncertainties were resolved by consensus after returning to the original report. Missing or unclear information was recorded as not reported rather than inferred. Data charting was therefore verified by the second reviewer, but it was not undertaken as two independent parallel extractions and no numerical agreement statistic was calculated Table 2.

**Table 2:** Data-charting domains

Domain	Information charted
Bibliographic characteristics	Author, year, title, journal/source, country or setting, and publication type.
Study characteristics	Aim, design, sampling approach, sample size, participant groups, age/sex where reported, competitive level, sport, and impairment group.
Classification exposure	Initial classification, reclassification, declassification, not-eligible decision, review status, protest/appeal, intentional misrepresentation, or implementation/change of a classification system.
Psychological and experiential evidence	Emotions, stress, anxiety, well-being, identity, belonging, motivation, self-efficacy, confidence, fairness, trust, dignity, stigma, uncertainty, perceived control, satisfaction, and behavioural responses.
Contextual influences	Communication, preparation, social support, classifier-athlete relationships, organisational practices, transparency, access, travel burden, and competitive or financial pressures.
Consequences and recommendations	Participation, performance, selection, career transition, retirement, withdrawal, coping, proposed support, and recommendations for classification practice.

### Critical appraisal

Formal methodological quality or risk-of-bias appraisal was not undertaken. JBI guidance indicates that critical appraisal is not mandatory in scoping reviews when the purpose is to map the range, characteristics, and gaps of a heterogeneous evidence base rather than estimate an intervention effect or grade certainty [21]. Accordingly, no study was excluded or numerically weighted on the basis of methodological quality. Study design, sample, data-collection methods, and reported methodological limitations were charted and considered when interpreting convergence, divergence, and the extent to which causal or comparative inferences could be supported. Interpretive claims were qualified when evidence was based on a single study, a cross-sectional association, or a retrospective account.

### Data synthesis

The evidence was synthesised using descriptive numerical mapping and inductive thematic synthesis [25]. Descriptive summaries were produced for publication year, study design, sport, participant composition, classification stage, and the psychological or experiential domains examined. Meta-analysis was not undertaken because the included studies differed substantially in design, participants, classification systems, constructs, and outcome reporting. For quantitative and mixed-methods studies, classification-related descriptive findings, associations, group comparisons, and qualitative components were charted alongside narrative evidence. Quantitative estimates were not pooled and were not treated as equivalent to qualitative testimony; they were mapped to a theme only when the reported result directly addressed the relevant psychological or experiential domain.

For the thematic synthesis, M.A. conducted the initial inductive coding at the level of reported experience, appraisal, response, or consequence. A working coding matrix was developed and iteratively refined as lower-order codes were compared across studies and grouped into descriptive categories. A.Ç.T. reviewed the code-to-category and category-to-theme assignments, challenged ambiguous cases, and contributed to interpretation. Disagreements were resolved by returning to the source report and reaching consensus. Higher-order theme boundaries were defined by their primary conceptual focus; where a finding legitimately crossed conceptual boundaries, a study could contribute to more than one theme. The thematic structure was repeatedly checked against the charted data and original reports until it adequately represented convergent, divergent, and neutral findings. Additional file 2 provides the evidence-to-theme matrix as an audit trail for these assignments.

### Stakeholder consultation

Stakeholder consultation was not undertaken. No Para athletes, coaches, classifiers, sport administrators, or external subject-matter experts were consulted specifically for this review. The synthesis therefore represents evidence reported in the included literature and should not be interpreted as a co-produced account of current stakeholder priorities.

### Use of generative artificial intelligence

OpenAI ChatGPT was used for English-language refinement, improvement of academic wording, and limited document-formatting assistance during manuscript preparation and revision. The literature searches, study screening, eligibility assessment, data extraction and charting, evidence synthesis, analyses, interpretation of findings, and development of the conclusions were conducted by the authors. All scientific decisions, citations, data, and final manuscript content were reviewed and approved by the authors, who take full responsibility for the integrity and accuracy of the work. ChatGPT was not used to make inclusion or exclusion decisions or to generate research data and was not listed as an author.

### Ethics

Ethics approval was not required because the review analysed information available in published and publicly accessible sources and did not involve the collection of new data from human participants.

## Results

### Study selection

**Fig. 1:**
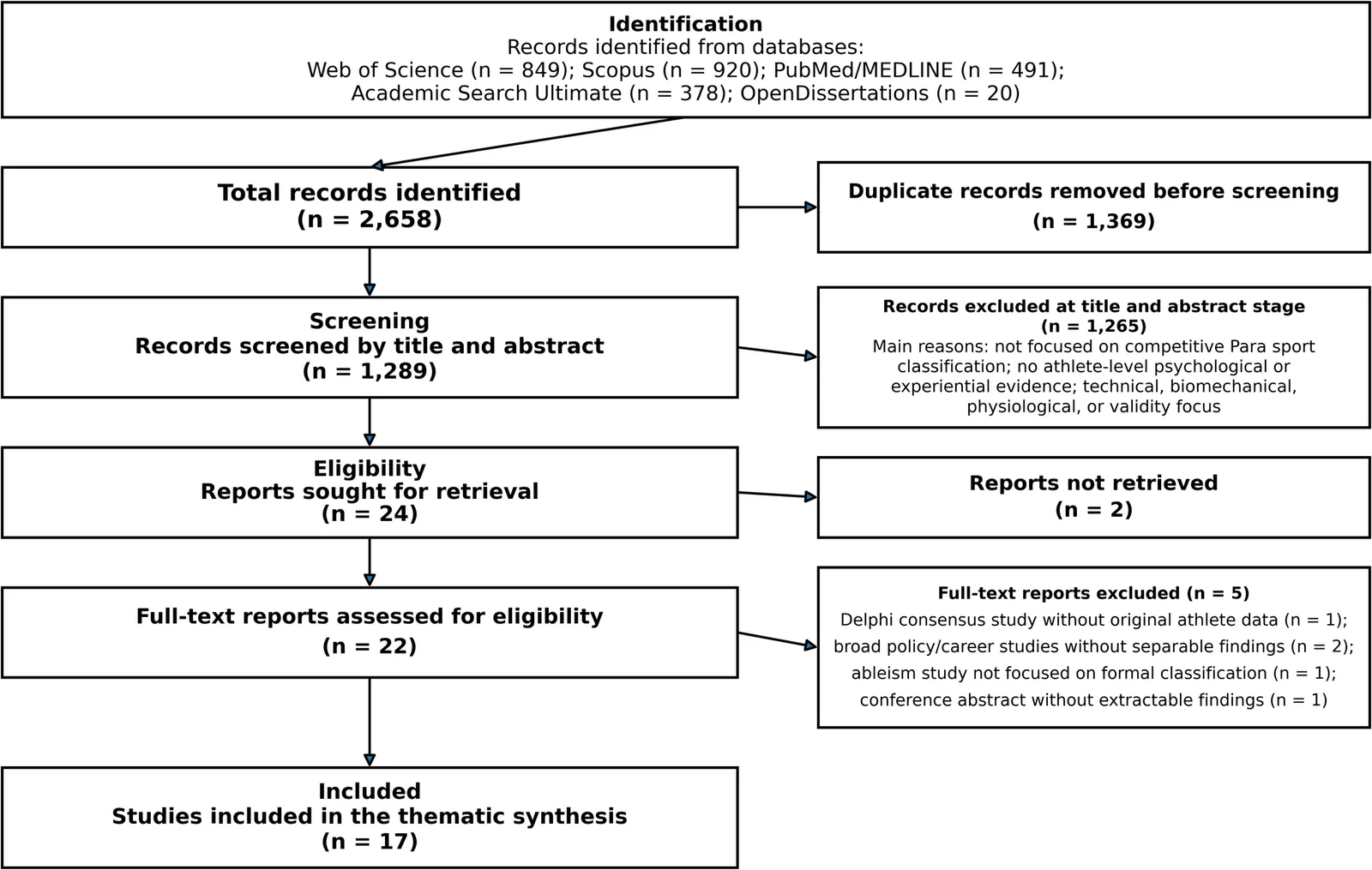
PRISMA 2020 flow diagram of study selection

Two reports could not be independently retrieved and were not incorporated into the synthesis Fig 1.

The database searches identified 2,658 records. After 1,369 duplicate records had been removed, 1,289 unique records underwent title-and-abstract screening. Of these, 1,265 were excluded because they did not examine competitive Para sport classification, did not report athlete-level psychological or experiential evidence, or were restricted to technical, biomechanical, physiological, or classification-validity questions. Twenty-four reports were sought for full-text assessment. Two reports could not be independently retrieved, leaving 22 full-text reports for eligibility assessment. Five reports were excluded at this stage: one Delphi consensus study without original athlete-experience data, two broad policy or career studies in which classification-specific psychological findings could not be separated, one study of ableism in swimming that did not focus on a formal Para sport classification process, and one conference abstract without extractable classification findings. Seventeen studies were included in the final synthesis Table 3.

**Table 3:** Study selection summary

Selection stage	Records/reports, *n*
Records identified from databases	2658
Duplicate records removed	1369
Unique records screened	1289
Records excluded after title–abstract screening	1265
Reports sought for retrieval	24
Reports not retrieved	2
Full-text reports assessed	22
Full-text reports excluded	5
Studies included in the synthesis	17

Counts are reported as records or reports, as appropriate

### Characteristics of the included evidence

The 17 included studies were published between 2003 and 2026, with 12 appearing from 2020 onwards. Ten studies were primarily qualitative, four used quantitative cross-sectional or survey designs, one used mixed methods, and two used participatory or multi-method evaluation designs. Seven studies addressed multiple sports or cross-sport classification systems, whereas 10 were sport-specific or focused on a small set of related sports. Wheelchair basketball was examined in three studies and Para swimming in two; the remaining evidence covered amputee football, Para alpine skiing, Para shooting, sitting volleyball, visually impaired cricket and football, remote classification across five Para sports, and wider multi-sport Para-athlete samples. Several studies combined athlete data with the perspectives of classifiers, coaches, support staff, parents, medical or technical personnel, and sport administrators. Athlete samples ranged from nine participants in an in-depth qualitative study to 222 athletes in a sitting-volleyball survey.

Most studies examined athletes’ experiences of an existing classification system, a proposed or newly developed sport-specific system, or the relationship between functional class and psychological or performance variables. Reclassification and loss of eligibility were examined directly in work on sport retirement and athlete well-being, while other studies addressed intentional misrepresentation, organisational perceptions of classification, or remote and hybrid access models. These classification exposures were not treated as interchangeable; their context was retained when findings were charted and interpreted.

### Classification contexts represented in the literature

The included literature represented distinct classification contexts rather than a single uniform exposure. Table 4 distinguishes studies involving athletes’ direct experiences of assessment, class allocation, reclassification, or declassification from studies evaluating existing or proposed systems, studies using functional class as an analytical exposure, work focused on intentional misrepresentation, and remote or hybrid classification. Initial classification and general system understanding were the most common contexts. Reclassification and declassification were rarely isolated from broader career experiences, while protest and appeal were discussed mainly as features of fairness and transparency rather than studied as independent psychological events. The synthesis therefore retained the classification context of each study when interpreting psychological or experiential findings.

**Table 4:** Characteristics of the 17 included studies

Study	Sport and participants	Design and classification context	Psychological or experiential focus
Engin et al. (2025) [26]	Amputee football Participants: 12 male amputee football players	Qualitative; interpretative phenomenological analysis Classification: Understanding and potential application of a classification system	Knowledge, fairness, acceptance, and implementation expectations
Douglas et al. (2024) [9]	Para alpine skiing; vision impairment Participants: 11 athletes	Qualitative interviews; reflexive thematic analysis Classification: Visual assessment procedures and sport classes	Eligibility, exclusion, trust, information, and intentional misrepresentation
Bosma and Van Yperen (2020) [10]	Wheelchair basketball Participants: 141 athletes	Quantitative cross-sectional study Classification: Functional classification points	Competitive anxiety, self-efficacy, and performance
Van Dornick and Spencer (2020) [7]	Para swimming Participants: 9 Para swimmers	Qualitative; interpretive description Classification: Athlete evaluation and sport-class allocation	Access, diversity, uncertainty, fairness, accuracy, and dignity
Bundon et al. (2018) [13]	Multiple Para sports Participants: Survey of 60 retired athletes; 13 qualitative interviews	Mixed methods Classification: Reclassification, loss of eligibility, and declassification	Trauma, powerlessness, shame, isolation, coping, and career transition
Lawson et al. (2023) [8]	Multiple Para sports Participants: 18 athletes and 8 classifiers	Qualitative interviews Classification: General classification process	Positive and negative experiences, system knowledge, communication, and mutual understanding
Gowans et al. (2025) [27]	Eleven Para sports Participants: 26 participants: 14 athletes, 7 support staff, and 5 classifiers	Qualitative interviews Classification: Intentional misrepresentation	Knowledge, pressure, motivation, trust, sanctions, and sporting integrity
Lee et al. (2026) [12]	Multiple elite Para sports Participants: 10 elite British athletes	Qualitative interviews Classification: Classification and reclassification	Perceived bias, uncertainty, loss of control, psychological turmoil, and psychological safety
Sales and Misener (2021) [28]	Para swimming Participants: 5 athletes and 7 parents	Qualitative; interpretative phenomenological analysis Classification: Classification within the athlete-development pathway	Development barriers, belonging, connection, and family experience
Allen et al. (2021) [29]	Para shooting; vision impairment Participants: 17 athletes, 7 coaches, and 4 classifiers	Multi-stakeholder evaluation Classification: New sport-specific vision-impairment classification system	Communication, trust, sport specificity, acceptance, and intentional misrepresentation
Fliess-Douer et al. (2003) [30]	Wheelchair basketball Participants: 59 adult male athletes	Quantitative cross-sectional survey Classification: Functional physical-impairment class	Task and ego goal orientations
Marszalek et al. (2019) [31]	Sitting volleyball Participants: 187 championship athletes and 35 league athletes	Quantitative survey Classification: Sitting-volleyball classification system	Satisfaction, perceived suitability, and views on system modification
Marques et al. (2012) [32]	Swimming, goalball, wheelchair rugby, and wheelchair basketball Participants: 4 athletes and 4 administrators	Qualitative semi-structured interviews Classification: Classification in the Brazilian Paralympic movement	Social tension, symbolic power, fairness, and athlete autonomy
Molik et al. (2017) [5]	Wheelchair basketball Participants: 79 athletes, 12 coaches, and 14 classifiers	Exploratory multi-stakeholder survey Classification: IWBF functional classification system	Satisfaction, system change, communication, and perceptions of cheating
Powis and Macbeth (2020) [6]	Visually impaired cricket and partially sighted football Participants: 16 male England VI cricketers and 7 male partially sighted footballers	Qualitative: ethnography, participant observation, and semi-structured interviews Classification: VI classes, combined classes and quotas, and intentional misrepresentation	Limited faith in classification, confusion, frustration, class-based social identity, stigma, social tension, and intention to leave sport
Miller et al. (2024) [16]	Multiple summer Para sports Participants: 21 current UK Para athletes (13 Paralympians and 8 internationally competitive athletes across 12 sports)	Qualitative semi-structured interviews; inductive thematic analysis Classification: classification and possible declassification as well-being stressors	Stress, lack of preparation, conflicting identities, well-being, and need for tailored support
Harrington and Misener (2025) [18]	Five Para sports in lower-resourced Commonwealth contexts Participants: athletes, classifiers, in-situ medical and technical personnel, and sport-organisation stakeholders	Participatory multi-method evaluation using capacity assessment, remote-classification trials, surveys, interviews, and focus groups Classification: remote and hybrid classification	Access, feasibility, time/travel/cost burden, communication, privacy, training implications, and participation pathways

*VI* Vision impairment, *IWBF* International Wheelchair Basketball Federation

### Thematic synthesis

Seven interrelated themes were identified. A study could contribute to more than one theme. Theme frequencies describe how often a domain was represented in the included literature; they do not indicate effect magnitude, methodological quality, or comparative importance. Across the evidence, psychological experiences were more often reported in relation to the conditions under which classification was conducted, explained, and accessed than to the numerical sport class alone.

#### Process knowledge, communication, and procedural intelligibility

Twelve studies showed that athletes’ understanding of classification was uneven and often dependent on informal explanations from coaches, classifiers, or more experienced athletes. Limited knowledge made it difficult to anticipate what would happen during assessment, interpret a decision, or understand the consequences of a class change. This problem was evident in established systems, proposed sport-specific systems, and remote-classification trials. Athletes evaluated communication not only by the amount of information provided but also by whether the explanation was timely, comprehensible, respectful, accessible, and connected to the demands of their sport. Clearer communication was associated with greater acceptance, whereas unexplained procedures allowed uncertainty and suspicion to persist [5–9, 12, 16, 18, 26–29].

#### Fairness, trust, and system integrity

Fairness, trust, and sporting integrity were reported in 11 studies. Athletes distinguished between technical fairness and procedural fairness. Technical fairness referred to whether impairment assessments and class boundaries reflected the functional requirements of the sport. Procedural fairness concerned consistency, transparency, respectful treatment, access to classification, and the opportunity to understand or challenge a decision. Trust weakened when tests appeared insufficiently sport-specific, when classifications were perceived as inconsistent, when access depended on substantial personal resources, or when athletes suspected intentional misrepresentation. These concerns affected relationships among competitors and confidence in the legitimacy of results [5–9, 12, 18, 26, 27, 29, 32].

#### Uncertainty, control, and emotional consequences

Eight studies identified uncertainty and loss of control as recurrent features of psychologically difficult classification experiences. Athletes described apprehension before assessment and concern about eligibility, class allocation, team selection, and future participation. The most severe accounts followed unexpected reclassification or loss of eligibility and included psychological turmoil, shame, powerlessness, isolation, and difficulty planning a sporting future. Confusion and limited confidence in the classification system were also reported alongside frustration and, in one visually impaired sport setting, an intention to leave competition. Quantitative evidence from wheelchair basketball did not show a direct association between functional class and competitive anxiety or self-efficacy [10]. Considered alongside the qualitative evidence, this finding suggests that psychological strain may be more closely related to appraisal, communication, uncertainty, and practical consequences than to class number alone [6, 7, 9, 12, 13, 16].

#### Identity, belonging, dignity, and social relations

Eight studies linked classification to athlete identity, belonging, dignity, and social relationships. Classification could legitimate an athlete’s place within Para sport and provide a route into meaningful competition. At the same time, athletes could feel required to demonstrate, explain, or defend their impairment in ways that threatened dignity. Classification labels also functioned as social identifiers and shaped expectations, team membership, perceived competence, and relationships with governing bodies. Reclassification or declassification was particularly disruptive when the athlete’s identity, social network, and future career were closely attached to the previous class or to continued eligibility [6, 7, 9, 12, 13, 16, 28, 32].

#### Satisfaction, acceptance, and perceived utility

Six studies demonstrated that classification was not experienced as inherently negative. Athletes expressed higher satisfaction when a system was viewed as sport-specific, understandable, accessible, and capable of grouping competitors meaningfully. Surveys in sitting volleyball and wheelchair basketball indicated broad acceptance of existing systems, while remote-classification trials were valued for reducing travel, time, and cost burdens. Support was nevertheless accompanied by requests for targeted modifications, clearer explanations, secure technology, appropriate privacy, and stronger safeguards against manipulation [5, 8, 18, 26, 29, 31].

#### Motivation, self-efficacy, behavioural responses, and performance

**Fig. 2:**
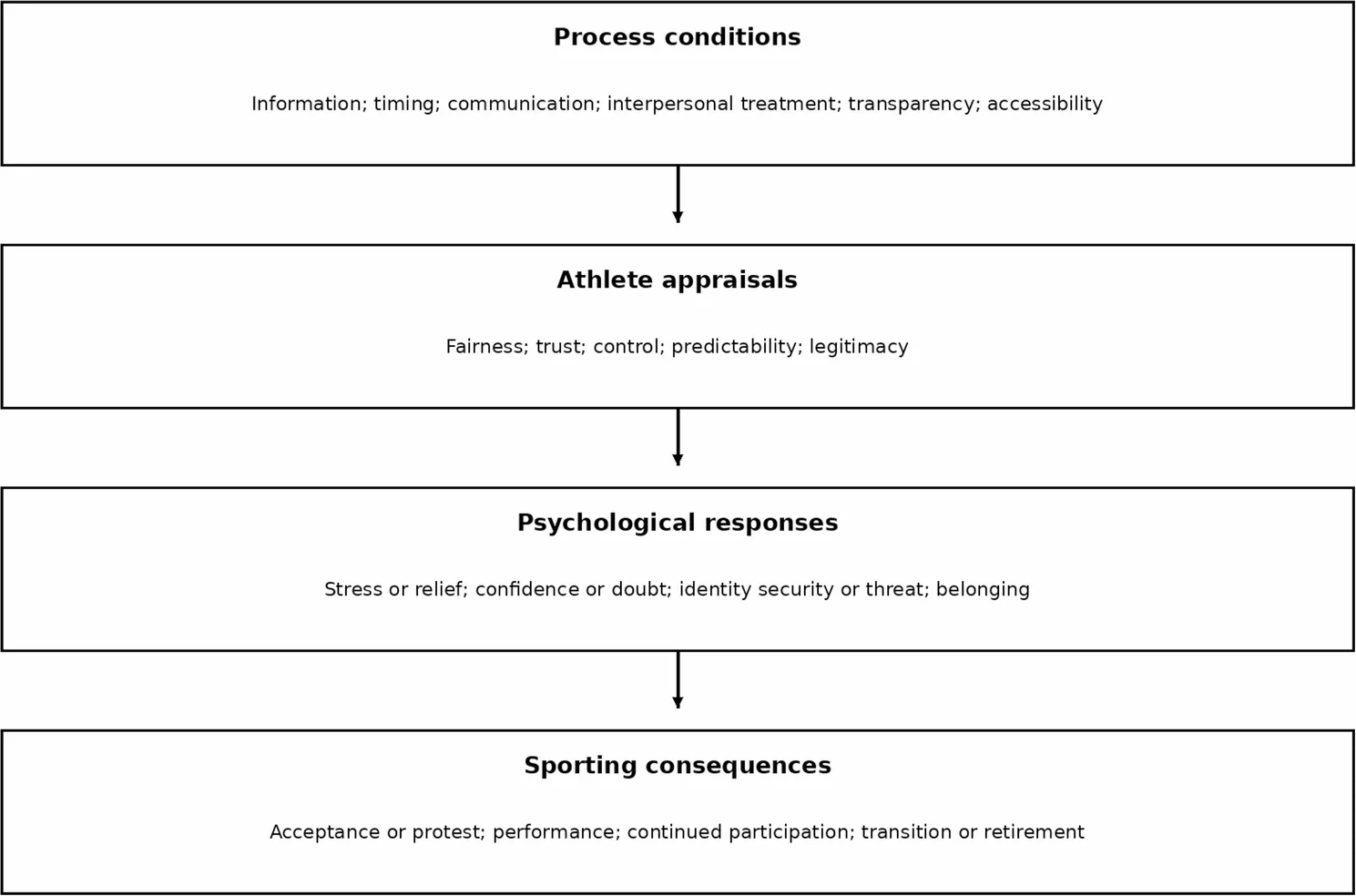
Evidence-informed pathway linking classification conditions to psychological and sporting consequences. Note. This pathway is an interpretive framework generated from the mapped evidence; it has not been prospectively validated as a causal model

Figure 2 Four studies examined motivation, self-efficacy, goal orientation, behavioural responses, or performance. Functional class was not consistently associated with competitive anxiety, self-efficacy, or task and ego orientation. Some performance differences were associated with class, but these findings did not establish a corresponding psychological disadvantage. Qualitative evidence indicated that competitive opportunity, financial incentives, pressure from the sport environment, perceived sanction risk, and class-based expectations were reported in relation to behaviour, including intentional misrepresentation, self-surveillance, and withdrawal intentions. The evidence remained too limited to determine how classification is associated with motivation or performance over time [6, 10, 27, 30].

#### Access, burden, and participation

Four studies showed that access to classification formed part of the athlete experience. In-person classification could require substantial travel, time, cost, administrative coordination, and access to qualified personnel, particularly in lower-resourced regions. Remote or hybrid processes were reported as reducing some of these burdens and helping athletes identify potential participation pathways. These benefits depended on secure technology, adequate private space, on-site support, clear communication, and recognition that remote assessment may complement rather than replace in-person classification. Classification access should therefore be understood as an organisational factor with potential relevance to participation rather than as an independently established determinant [7, 16, 18, 28] Table 5.

**Table 5:** Summary of the thematic synthesis

Theme	Studies, *n*	Synthesis
Process knowledge, communication, and procedural intelligibility	12	Accessible explanations and respectful communication improved predictability and acceptance; limited knowledge increased uncertainty and dependence on informal sources.
Fairness, trust, and system integrity	11	Trust depended on sport-specific tests, consistent decisions, transparency, accessible procedures, and credible review routes; suspected misrepresentation undermined confidence.
Uncertainty, control, and emotional consequences	8	Eligibility uncertainty and unexpected class changes produced apprehension, loss of control, and, in severe cases, shame, isolation, psychological turmoil, and withdrawal intentions.
Identity, belonging, dignity, and social relations	8	Classification shaped legitimacy, dignity, class-based social identity, team membership, and sporting identity; reclassification or declassification could destabilise these relationships.
Satisfaction, acceptance, and perceived utility	6	Satisfaction was higher when systems were understandable, accessible, and meaningful for competition, although athletes still requested targeted revisions and safeguards.
Motivation, self-efficacy, behavioural responses, and performance	4	Class was not consistently associated with anxiety, self-efficacy, or goal orientation; pressures, expectations, and incentives could influence behaviour.
Access, burden, and participation	4	Travel, cost, infrastructure, privacy, and classifier availability shaped access; remote or hybrid processes reduced burden when appropriate safeguards and support were present.

A study could contribute to more than one theme. Frequencies describe the number of studies in which a theme was represented; they are not effect estimates, quality weights, or rankings of evidential strength and may be influenced by study design, sample size, interview focus, reporting depth, and coding decisions

### Evidence gaps

The principal evidence gap was the scarcity of prospective and longitudinal research. Most included studies were qualitative, retrospective, or cross-sectional, and no study followed athletes prospectively from before classification or reclassification through the decision and subsequent competition or transition. No included study prospectively evaluated a classification-specific support strategy such as education, psychological preparation, decision debriefing, remote-classification support, or post-classification care. Psychological outcomes were usually embedded within broader accounts of experience rather than assessed with classification-specific validated measures.

The literature was concentrated in a small number of sports, particularly wheelchair basketball and Para swimming, while some impairment groups and classification systems were represented by only one study. Sex-disaggregated findings, youth experiences, intellectual-impairment sport, and evidence from lower-resourced settings were limited. Protest, appeal, review status, repeated reclassification, and remote or hybrid classification were rarely investigated as distinct psychological events. Given the limited range of countries, sports, impairment groups, and competitive contexts represented, transferability to the broader international Para sport population should not be assumed.

Two reports that met the title-and-abstract criteria could not be independently retrieved and were not incorporated into the definitive thematic synthesis. Their abstracts indicated potential relevance to Para-surfing satisfaction and virtual Para-rowing classification. Their absence may have reduced the representation of satisfaction, remote assessment, and access-related experiences.

### Summary of findings

The available evidence suggests that psychological experiences of classification are reported in relation to both the decision itself and the way the process is organised and accessed. Information, communication, interpersonal treatment, timing, transparency, accessibility, and practical burden were associated with athletes’ appraisals of fairness, trust, control, and legitimacy. Reported responses included distress, relief, confidence, identity threat, stronger belonging, and altered participation, with possible implications for acceptance, protest, performance, continued involvement, and career transition. These relationships should be interpreted as descriptive and associative rather than as a validated causal pathway.

## Discussion

### Principal findings

This review brought together 17 studies examining how athletes encountered classification and how those encounters were reported in relation to psychological experience, behaviour, participation, and career development. The literature remained small and methodologically diverse, but recurrent patterns were evident. Athletes’ accounts were associated not only with the sport class assigned but also with the information available before assessment, the conduct of the evaluation, the explanation of the decision, the accessibility of the process, and the practical consequences that followed. Communication, fairness, trust, uncertainty, identity, belonging, and access were therefore interpreted as connected elements of the classification experience rather than isolated causal determinants [5–10, 12, 13, 16, 18, 26–32].

The most disruptive accounts concerned situations in which athletes could not predict the procedure, did not understand the basis of a decision, believed that the assessment did not reflect the functional demands of their sport, or faced substantial barriers to obtaining classification. Unexpected reclassification or loss of eligibility was reported in connection with particularly difficult experiences because these decisions could alter selection prospects, team roles, funding, sporting identity, and continued access to competition [7, 9, 12, 13, 16]. Positive and neutral experiences were also present. Athletes more often described acceptance when the system appeared sport-specific, the rationale was intelligible, access was feasible, and classifiers communicated with consistency and respect [5, 8, 18, 26, 29, 31]. These patterns support interpreting classification as a technical decision delivered through an organisational and interpersonal process, while not establishing a causal effect of any single process feature.

### Classification as an organisational and psychological demand

The synthesis extends research that identifies classification as a distinctive organisational demand in Para sport [4]. Elite athletes already operate within environments characterised by selection pressure, performance evaluation, injury risk, public scrutiny, and uncertainty. Para athletes may encounter additional demands associated with accessibility, disability management, equipment, stigma, and dependence on sport organisations [14–16]. Classification differs from many other organisational stressors because it determines the conditions under which an athlete is permitted to compete and may itself be difficult or costly to access. Its psychological significance arises from the combination of evaluative exposure, material consequence, and organisational dependence.

The absence of a consistent association between functional class and competitive anxiety or self-efficacy in wheelchair basketball is important [10]. It indicates that class number alone is unlikely to explain psychological response. The wider evidence instead points to appraisal of the process as a potentially important correlate: whether the athlete considers the decision accurate, predictable, respectful, accessible, and open to review. This interpretation accords with broader elite-athlete mental health guidance, which places organisational culture, stress exposure, interpersonal relationships, and access to support alongside individual vulnerability [33]. Distress surrounding classification should not automatically be treated as evidence of a mental disorder. The available studies do not establish that classification causes clinical symptoms; rather, sustained uncertainty, humiliation, exclusion, inaccessible procedures, or involuntary career disruption were described in contexts where psychological difficulty was also reported. Classification policy and athlete mental health provision may therefore warrant coordinated prospective evaluation.

### Fairness, communication, and trust

Fairness was the most consistent interpretive lens across the included studies. Athletes evaluated both the outcome and the manner in which it was reached. Organisational justice research distinguishes distributive justice, procedural justice, interpersonal justice, and informational justice [34]. The same distinctions were evident in the classification literature. Distributive concerns addressed the class allocated and its competitive implications. Procedural concerns addressed the consistency of testing, practical access to assessment, and the opportunity to question a decision. Interpersonal concerns addressed dignity and respectful treatment. Informational concerns addressed the adequacy and clarity of explanations [5–9, 12, 18, 26, 29, 32].

Technical improvements remain necessary, particularly where impairment tests do not adequately represent sport-specific activity. Technical validity alone, however, cannot secure trust. A scientifically defensible system may still be experienced as illegitimate when athletes receive insufficient preparation, encounter unexplained delays, cannot afford or reach classification opportunities, or cannot understand how evidence was translated into a class decision. Conversely, clear communication and easy access cannot compensate for an assessment that lacks sport specificity. Trust depends on scientific credibility, procedural integrity, and realistic access.

Suspected intentional misrepresentation further complicates this relationship. Rumours of manipulation affected confidence in competitors and classifiers even when athletes had no direct evidence of wrongdoing [6, 9, 27, 29]. Federations may consider communicating the evidential threshold, investigative procedure, and consequences associated with intentional misrepresentation in ways that do not encourage informal surveillance among athletes. The acceptability and effects of such communication should be evaluated. Transparent governance may reduce speculation while protecting athletes from accusation based on appearance, impairment stereotype, or misunderstanding of fluctuating function.

### Uncertainty, identity, and career consequences

Uncertainty was most often described as psychologically difficult when athletes perceived limited control over decisions with immediate career consequences. Initial classification may create apprehension, whereas reclassification, declassification, and ‘Not Eligible’ decisions can threaten an established sporting role. The affected athlete may lose a class in which technical skills, team relationships, and performance expectations have developed over years. Classification labels may also operate as social identities, shaping expectations and hierarchies within teams [6]. In the included accounts, such decisions were sometimes experienced as both regulatory events and involuntary career transitions [11–13, 16].

Research on athletic retirement supports the importance of voluntariness, identity, social networks, and preparation. Strong athletic identity can increase vulnerability to anxiety during the transition out of sport [35], while reviews of retirement evidence describe identity disruption, loss of social connection, and uncertainty about future roles [36, 37]. These processes may be relevant when classification accelerates retirement or requires a move to another sport. Future research should evaluate whether support offered before a foreseeable eligibility change improves understanding, coping, or transition outcomes. Candidate support may include time to consider possible outcomes, alternative competitive pathways, and access to psychological or career guidance.

Dignity also requires attention. Several studies described the burden of repeatedly demonstrating impairment or explaining why an athlete belongs within Para sport [7, 9, 11, 12]. Assessment may involve sensitive medical and functional information and can position the athlete as an object of scrutiny rather than an informed participant. Privacy, consent, accessible explanation, and access to an athlete support person are plausible elements of athlete-centred practice, but their specific value within classification settings requires prospective evaluation.

### Implications for classification practice and athlete support

The findings suggest that a staged athlete-centred approach to classification merits development and prospective evaluation. Before assessment, candidate procedures could include concise, accessible information on eligibility criteria, assessment components, possible outcomes, review status, protest and appeal routes, confidentiality, expected timelines, and available support. Information could be adapted for different communication and access needs, and future studies should examine whether a named contact reduces dependence on informal explanations from peers or coaches.

During assessment, candidate procedures for evaluation include clear explanation of the purpose of each component, identification of who is present and why, consistent standards of interpersonal conduct, and a route for athletes to request clarification without concern that doing so will prejudice the decision. Remote or hybrid procedures may reduce travel and cost burdens in appropriate circumstances, but their acceptability and practical consequences should be evaluated alongside secure technology, privacy, in-situ assistance, and management of error risk [18].

Classification-specific psychological interventions have not yet been evaluated. Recommendations concerning support should therefore be treated as evidence-informed, practice-oriented hypotheses rather than established effects. Candidate approaches for prospective evaluation include decision debriefing, access to a support person, and referral when athletes report marked distress or face involuntary career change. The International Olympic Committee Sport Mental Health Assessment Tool and Recognition Tool provide general routes for identifying athletes who may require further assessment [38]; any use in classification settings would require contextual adaptation and evaluation.

The evidence also warrants evaluation of whether classifier education should include communication under uncertainty, disability-sensitive practice, procedural fairness, management of disagreement, and recognition of distress in addition to technical competency. Athlete representatives could contribute to the development and review of educational materials, assessment environments, remote-classification procedures, and appeal information. Although stakeholder consultation was not undertaken in this review, the included literature repeatedly indicated that athlete voice was associated with whether classification was understood as legitimate and workable [5, 8, 16, 18, 26, 29, 31].

### Research priorities

The evidence base requires stronger longitudinal and comparative designs. Prospective studies should assess athletes before classification, immediately after the decision, and during subsequent competition or transition. Such designs could help distinguish anticipatory stress from responses reported after the decision and establish whether reactions persist, resolve, or intensify over time. Repeated assessment is particularly important for review status, progressive impairments, and sports in which reclassification occurs frequently.

Measurement also requires development. Current studies often embedded classification within broader interviews or relied on general measures of anxiety, self-efficacy, or satisfaction. A classification-specific instrument should assess procedural clarity, informational adequacy, interpersonal treatment, perceived accuracy, trust, control, identity threat, and anticipated sporting consequence. Development should involve athletes from different sports, impairment groups, competition levels, sexes, ages, and cultural settings. Quantitative measurement should complement, not replace, qualitative accounts of complex or contested decisions.

Prospective evaluation of supportive strategies is absent. Candidate approaches include pre-classification education, athlete advocates or support persons, structured decision debriefing, classifier communication training, psychological preparation for review status, remote-classification support, and transition planning after loss of eligibility. Studies should assess feasibility and acceptability before testing effects on knowledge, trust, distress, access, appeal use, and continued participation. Protest and appeal experiences, youth pathways, intellectual-impairment sport, remote and hybrid classification, and lower-resourced settings remain especially underrepresented Table 6.

**Table 6:** Research-priority matrix for future classification research

Population or context	Classification stage	Psychological or experiential priority	Recommended study design
Youth athletes and families	Initial/developmental classification	Understanding, uncertainty, identity, family support	Longitudinal mixed-method cohort
Athletes with intellectual impairment	Initial classification and reclassification	Comprehension, autonomy, dignity, support needs	Accessible participatory mixed-method study
Athletes facing reclassification or Not Eligible decisions	Pre-decision, decision, and transition	Distress, perceived control, identity, career transition	Prospective repeated-measures cohort
Athletes using review, protest, or appeal routes	Post-decision review and appeal	Procedural fairness, trust, psychological safety	Prospective process evaluation
Remote/hybrid and lower-resourced settings	Access and assessment	Burden, privacy, acceptability, trust	Comparative implementation and feasibility study
Diverse sex, cultural, and sport contexts	Repeated classification across the pathway	Differential experiences, belonging, well-being	Multicentre longitudinal comparative study

### Strengths and limitations

The review used a broad search strategy across five complementary information sources, supplemented by psychological sensitivity searches and known-item checks. The intentionally broad strategy reduced the risk of missing studies that described experience, fairness, access, or career consequences without using explicit psychological terminology. Two reviewers independently completed title-and-abstract and full-text screening, database-specific search strategies and exclusion decisions were documented, and the completed data charting was verified by the second reviewer. The addition of studies concerning visually impaired sport, Para-athlete well-being, and remote classification expanded the synthesis beyond sport-class allocation alone.

Several limitations affect interpretation. SPORTDiscus, APA PsycINFO, and Embase were not searched because access was unavailable through either institutional subscriptions or personal access routes available to the authors during the review period. Their omission may have introduced database-related selection bias, particularly for sport-psychology and disability-focused literature. The broad searches, sensitivity checks, and known-item tests reduced but could not eliminate the possibility that relevant studies were missed. No language filter was applied at the search stage, but the review did not use a formal professional-translation or multilingual-reviewer procedure, so coverage of non-English full texts may be incomplete. Two reports that met the title-and-abstract criteria could not be independently retrieved. The internal review plan was not prospectively registered or publicly time-stamped, and stakeholder consultation was not undertaken. Data charting was initially completed by one reviewer and verified by the second rather than independently double-charted, leaving some possibility of extraction or interpretative bias. Formal critical appraisal was omitted in keeping with the mapping purpose of the scoping review; consequently, the synthesis does not grade certainty or compare methodological quality. The small number of studies, limited representation of sports, countries, impairment groups, and classification stages, and predominance of qualitative, retrospective, or cross-sectional designs restrict conclusions about prevalence, causation, transferability, and differences between classification systems. Theme frequencies indicate representation within the literature and should not be interpreted as effect estimates or rankings of evidential strength.

## Conclusions

Para sport classification has psychological significance because it regulates access to competition while requiring athletes to undergo assessment and organisational decision-making. Across the included studies, psychological experiences were reported in relation to both classification decisions and the conditions under which those decisions were delivered. Clear information, respectful communication, sport-specific assessment, transparent reasoning, and credible review routes were commonly associated with trust and acceptance, whereas uncertainty, perceived inconsistency, limited control, and unexpected loss of eligibility were reported in the most disruptive accounts. These patterns are descriptive and associative and should not be interpreted as evidence of causation.

Athlete-centred classification does not require less scientific rigour; it requires technical rigour to be delivered through procedures that protect dignity, are realistically accessible, and acknowledge sporting consequences. Classification education, decision debriefing, remote-access safeguards, psychological referral pathways, and transition planning should be regarded as evidence-informed priorities for prospective evaluation rather than established effective practices. Future research should follow athletes longitudinally, evaluate supportive strategies, and compare experiences across underrepresented populations and classification stages. The evidence-informed pathway proposed in this review remains interpretive and hypothesis-generating, not a prospectively validated causal model.

## Supplementary Information

Additional file 1. Complete database search strategies. Description: Database-specific search dates, platforms/interfaces, fields, exact record counts, complete search strings, and supplementary psychological sensitivity-search details.

Additional file 2. Evidence-to-theme matrix for the 17 included studies. Description: Study context, classification exposure, key psychological or experiential evidence, thematic assignments, and classification-context descriptors.

## Data Availability

All data analysed during this study were obtained from publicly available published studies. The data supporting the findings of this review are included in the article and its supplementary information files.

## Abbreviations

IPC: International Paralympic Committee
IWBF: International Wheelchair Basketball Federation
JBI: Joanna Briggs Institute
PERSiST: Implementing PRISMA in Exercise, Rehabilitation, Sport Medicine and Sports Science
PRISMA: Preferred Reporting Items for Systematic Reviews and Meta-Analyses
PRISMA-ScR: PRISMA extension for Scoping Reviews
PRISMA-S: PRISMA extension for reporting literature searches
SMHAT-1: Sport Mental Health Assessment Tool 1
SMHRT-1: Sport Mental Health Recognition Tool 1
